# Lipid Rafts and Dopamine Receptor Signaling

**DOI:** 10.3390/ijms21238909

**Published:** 2020-11-24

**Authors:** Victor J. Martinez, Laureano D. Asico, Pedro A. Jose, Andrew C. Tiu

**Affiliations:** 1 Department of Medicine, Einstein Medical Center Philadelphia, Philadelphia, PA 19141, USA; tiuandre@einstein.edu; 2 Division of Renal Diseases & Hypertension, Department of Medicine, The George Washington University School of Medicine & Health Sciences, Washington, DC 20052, USA; lasico@email.gwu.edu (L.D.A.); pjose@mfa.gwu.edu (P.A.J.); 3 Department of Pharmacology/Physiology, The George Washington University School of Medicine & Health Sciences, Washington, DC 20052, USA

**Keywords:** lipid rafts, dopamine receptor, signaling, G protein

## Abstract

The renal dopaminergic system has been identified as a modulator of sodium balance and blood pressure. According to the Centers for Disease Control and Prevention, in 2018 in the United States, almost half a million deaths included hypertension as a primary or contributing cause. Renal dopamine receptors, members of the G protein-coupled receptor family, are divided in two groups: D1-like receptors that act to keep the blood pressure in the normal range, and D2-like receptors with a variable effect on blood pressure, depending on volume status. The renal dopamine receptor function is regulated, in part, by its expression in microdomains in the plasma membrane. Lipid rafts form platforms within the plasma membrane for the organization and dynamic contact of molecules involved in numerous cellular processes such as ligand binding, membrane sorting, effector specificity, and signal transduction. Understanding all the components of lipid rafts, their interaction with renal dopamine receptors, and their signaling process offers an opportunity to unravel potential treatment targets that could halt the progression of hypertension, chronic kidney disease (CKD), and their complications.

## 1. Introduction

The classical definition of a cell membrane is a semipermeable membrane, composed of a lipid bilayer mixed with proteins, that surrounds the cytoplasm and nucleoplasm. The cell membrane demarcates the inner from the outer cellular environment. The phospholipids and sphingolipids in the cell membrane function as building blocks because of their aggregative properties. These phospholipids consist of a hydrophilic head and hydrophobic tails. The hydrophobic tails interact with each other whereas the hydrophilic heads face the inner cellular and extracellular space. In 1997, the concept of rigid lipids and protein mixture changed when the formalized hypothesis on lipid rafts was published as a concept paper on “functional rafts” [1]. Further research has helped in the understanding of the compartmentalization of cell membranes into lipid and non-lipid raft microdomains [2]. Many receptors are synthesized intracellularly and targeted to these microdomains, which assist receptors in numerous cellular processes including, but not limited to, ligand affinity and cellular signal transduction. Several studies involving dopamine receptors have shown the delicate interaction between dopamine receptors and lipid rafts, as well as the need of functional lipid rafts so that dopamine receptors can operate properly [2].

The renal dopaminergic system plays a key role in the control of blood pressure, in part by regulating renal sodium transport [3]. In 2018, almost half a million deaths included hypertension as a primary or contributing cause. Hypertension is caused, in part, by expansion of the extracellular fluid, because of sodium retention due to an inability to excrete a sodium load [3,4,5]. For this reason, the study of lipid rafts and dopamine receptors could offer therapeutic targets related to essential hypertension. The activation of dopamine receptors has been shown to participate in the reduction of reactive oxygen species production and inflammation, which cause the progression of chronic kidney disease (CKD) [4,5]. Thus, further research on this topic can open new targets to stop the progression of hypertension, CKD, and their complications.

## 2. Definition of Lipid Rafts, Structure, Function, and Associated Components

**Figure 1:**
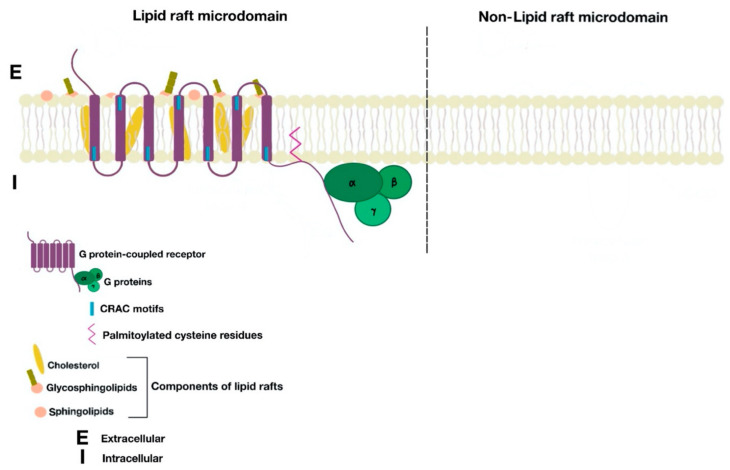
Cell membrane partitions into lipid raft and non-lipid raft microdomains.

Lipid rafts (Figure 1) are dynamic and shifting assemblies of sphingolipids, cholesterol, glycosphingolipids, and proteins forming platforms or lipid microdomains for the organization and dynamic contact of molecules involved in several molecular and cellular processes such as ligand affinity, effector specificity, signal transduction, membrane sorting, and receptor trafficking and recycling. Cholesterol molecules rest between the hydrophobic tails of phospholipids; next to them, many proteins serve as anchor units for other proteins. The organization of certain groups of proteins allows optimal function, as well as specification of cellular signal transmission by granting effective interaction between proteins and avoiding interference of opposite signaling pathways [2,6]. Lipid rafts are located in cell surface membranes, as well as in intracellular membranes. These allow the coordination of cellular processes, which in turn affect bioactivity of many receptors and the cell itself [2].

**Figure 2:**
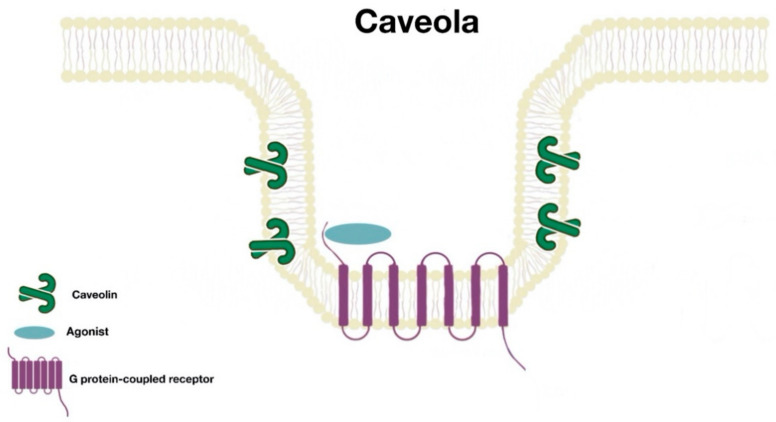
Caveola structure.

Many proteins are associated with lipid rafts; one of the best characterized proteins are the caveolins. These form caveolae (“little caves”). Caveolae (Figure 2) are scaffolding proteins that assemble within lipid raft domains to form flask-shaped invaginations of the plasma membrane. These platforms allow protein–protein, lipid–lipid, and protein–lipid interactions and regulate numerous cellular processes (signal transduction, receptor trafficking, recycling, etc.). There are three caveolin isoforms (Cav1, Cav2, and Cav3). However, Cav1 is the isoform that is present in most tissues and is enough for the formation of caveolae [7].

The function of surface membrane receptors is determined by their intracellular trafficking pathway. These receptors are produced intracellularly and their trafficking to the plasma membrane is needed for the attachment to the extracellular agonist or antagonist. Agonist-induced activation of the receptor is followed by its subsequent internalization and re-insertion to the plasma membrane (desensitization and resensitization of the receptor), ultimately controlling the function and activity of the receptor [7,8,9,10,11].

G protein-coupled receptors (GPCRs) are the largest superfamily of mammalian surface membrane receptors that work as signaling proteins. These are involved in diverse physiological processes by binding to ligands and transducing intracellular signaling pathways via activation of G proteins [9,10,11], i.e., G protein subunits α, β, and γ. The structure of GPCRs features the presence of seven transmembrane α-helical molecules, which are connected by three extracellular and three intracellular loops [10].

Surface membrane protein receptors, including GPCRs, undergo a trafficking process with constant co-translational and post-translational changes before arriving at the plasma membrane. As with many cellular proteins, GPCRs, following their synthesis, reside in the endoplasmic reticulum (ER) where they undergo assembly, folding, and packaging. The GPCRs temporarily reside in endoplasmic reticulum-derived COPII (coat protein complex II) transport vesicles, before exiting the ER into the Golgi apparatus. There, the GPCRs undergo further modifications, such as the S-palmitoylation of cysteine residues. At the Golgi edge, the GPCRs are boxed again into vesicles, into the endosomal pathway, which targets the receptors to the plasma membrane. In the plasma membrane, the GPCRs mature and become functional. Some subsets of GPCRs are targeted to specific regions of the plasma membrane, such as the lipid rafts [11].

**Figure 3:**
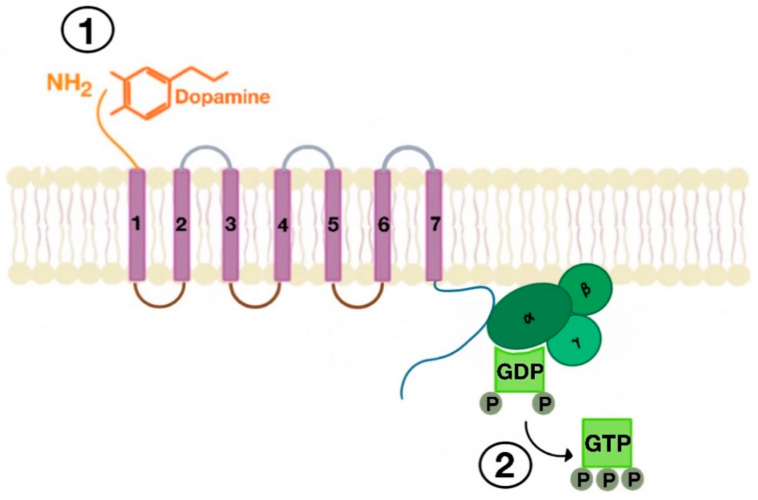
Agonist-induced stimulation of G protein-coupled receptors (GPCRs) (e.g., dopamine receptor (1) and phosphorylation of GDP to GTP on the Gα subunit of the G protein (2)).

**Figure 4:**
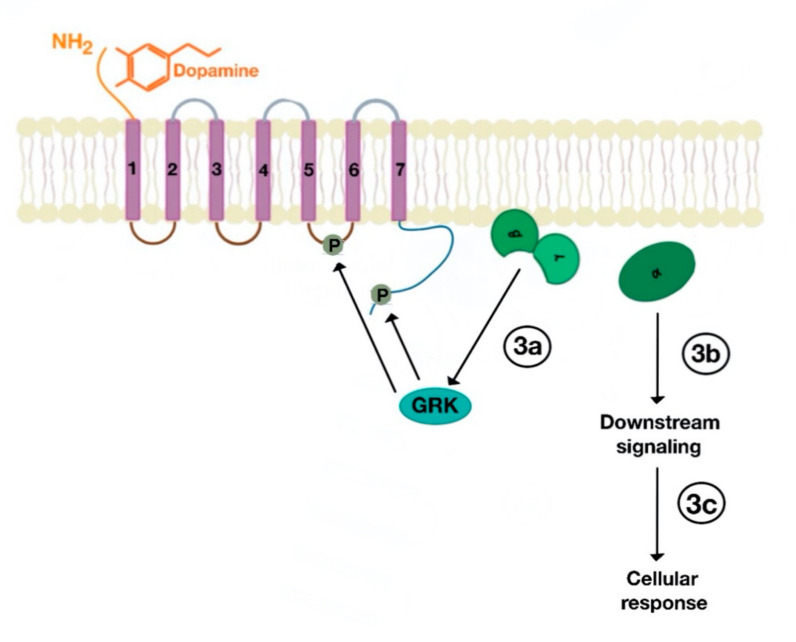
Uncoupling of Gα subunit from Gβγ subunit, followed by activation GPCR kinases (GRKs), which phosphorylate amino acids in the third intracellular loop of the transmembrane segments and C-terminal tail (3a). The uncoupled Gα subunit activates downstream cellular (3b) and subsequent cellular response (3c).

**Figure 5:**
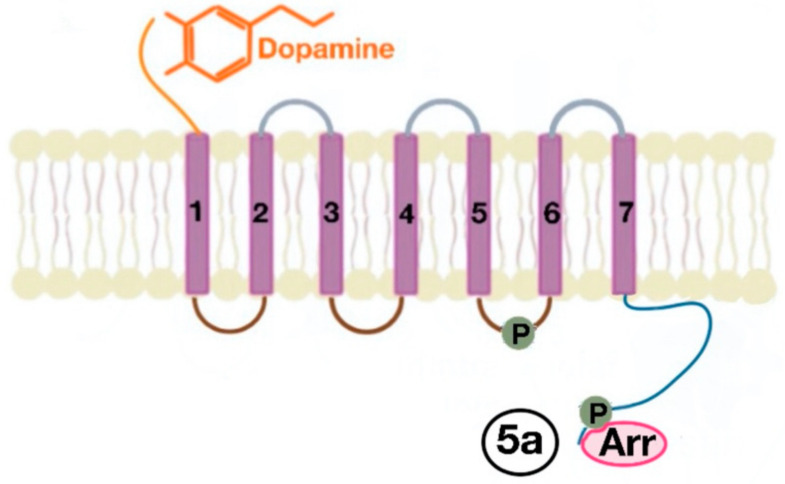
The phosphorylation of amino acids in the third intracellular loop of the transmembrane segments and C-terminal tail leads to the activation of β-arrestins (5a).

There are many motifs of proteins in the GPCRs that interact with the plasma membrane and its associated cholesterol. These motifs carry a cholesterol recognition/interaction amino acid consensus or CRAC motif. The CRAC motif helps anchor the Cav isoforms to the plasma membrane [12] as well as other plasma proteins, including GPCRs. Once the GPCR is targeted to a lipid raft, it can be successfully stimulated by interacting with its ligand (Figure 3). The agonist occupation of GPCRs, as in dopamine receptors, results in a conformational change promoting the exchange of GDP (guanosine diphosphate) to GTP (guanosine triphosphate) on the Gα subunit of the G protein, leading to two actions (Figure 4). First, the uncoupling of the G proteins from the GPCR, and second, the uncoupling of the Gα subunit from the Gβγ subunit. Finally, the Gα subunit stimulates or inactivates downstream signaling processes, depending on its conformational and functional characterization, Gαs being stimulatory or Gαi being inhibitory of adenylyl cyclase activity. The remaining Gβγ subunit activates GPCR kinases (GRKs), which phosphorylate amino acids in the third intracellular loop of the transmembrane segments and C-terminal tail of the receptor, leading to the activation of cytosolic proteins known as arrestins [13,14] (Figure 5). The activation of GRKs and β-arrestins ensures the removal of the GPCR from the cell surface, leading to its desensitization.

The β-arrestins, originally discovered for their inhibitory part in some receptor signaling pathways, have been proven to work as critical controllers of agonist-induced endocytosis and ubiquitination of plasma receptors, including GPCRs. Therefore, β-arrestins play a pivotal role in the trafficking path of endocytosed GPCRs [13,14].

**Figure 6:**
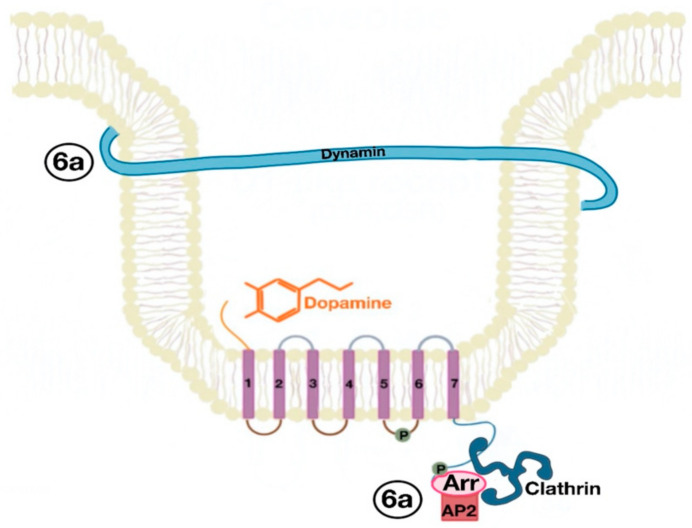
Dynamin and clathrin associate with the GPCR (dopamine receptor) and mediate its endocytosis by means of the adaptor protein 2 (AP2) (6a).

**Figure 7:**
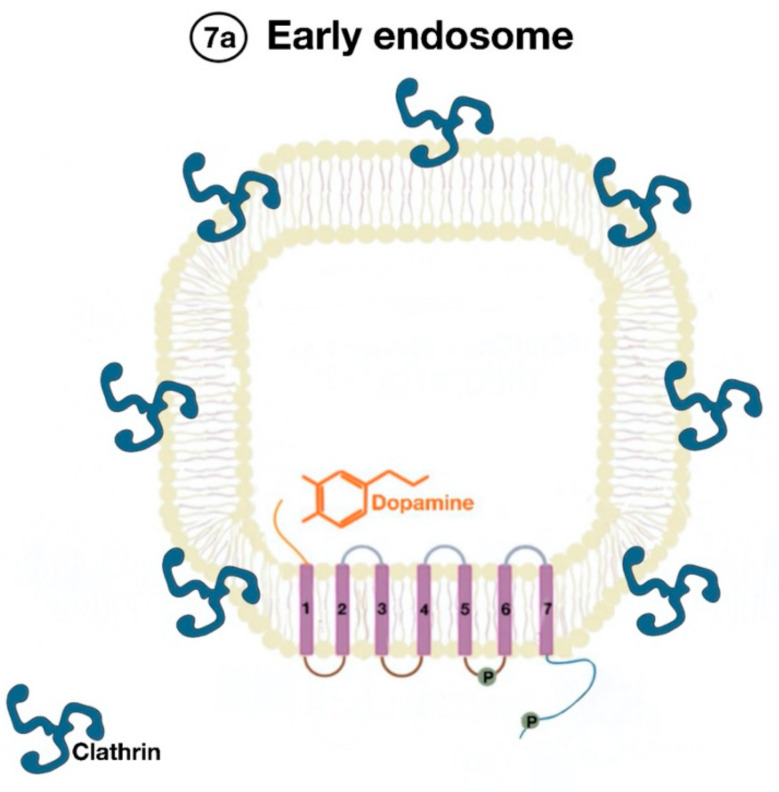
Endocytosed GPCR (dopamine receptor) is packaged in an early endosome (7a).

**Figure 8:**
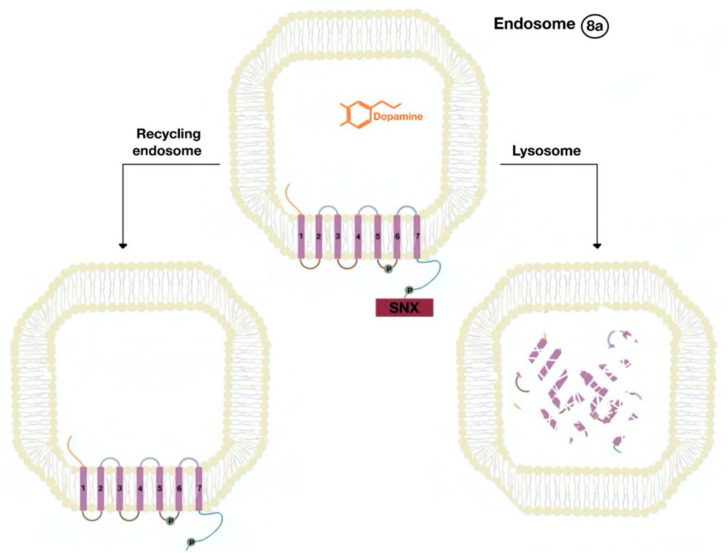
The endocytosed GPCR (dopamine receptor) binds to sorting nexin (SNX) that sends the GPCR either to a recycling endosome or lysosome (8a). Receptors targeted to lysosomes are degraded.

The β-arrestins direct sequestration of GPCRs via a dynamin-dependent, clathrin-mediated endocytosis. This process takes effect through adaptor protein 2 (AP2) complexes, which in turn begin clathrin-coated pit assembly (Figure 6). As soon as the GPCR is internalized, it is packaged in early endosome (Figure 7), where the GPCR is dephosphorylated by protein phosphatase 2A, in the case of the D_1_R [14]. Then, sorting nexins (SNXs) distribute the GPCR to different destinations [15]. From the endosome, the receptor is either sent to fast recycling endosome and subsequently to the lipid raft in the plasma membrane, or to lysosomes (Figure 8) for eventual degradation.

**Figure 9:**
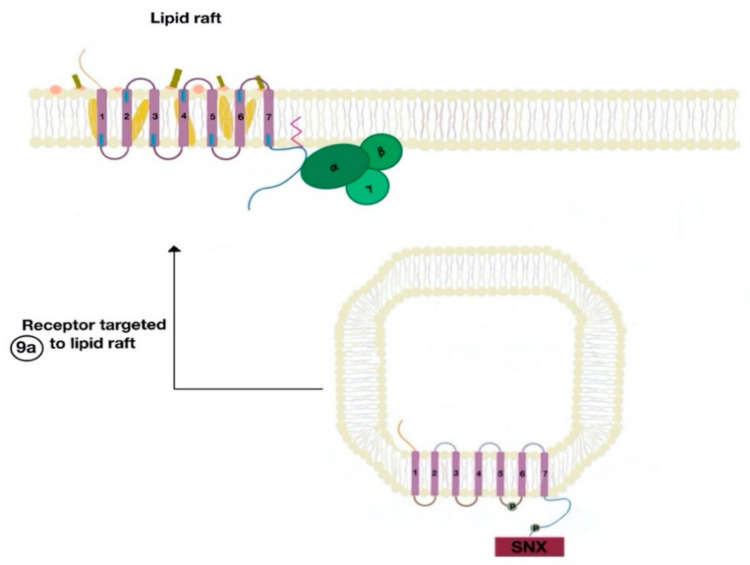
The resensitized GPCR (dopamine receptor) is targeted to the lipid raft microdomain by SNX (9a) and is ready to bind to a new agonist (9a).

The SNXs are a diverse group of membrane-linked phosphoinositide-binding proteins that regulate cellular protein trafficking by controlling all aspects of the endocytic pathway, including endocytosis, endosomal sorting, and endosomal signaling [16]. The SNX either sorts the endocytosed and dephosphorylated/resensitized GPCR into the fast recycling endosomes, targeting it to lipid rafts (Figure 9), or send such receptor to late endosomes, targeting it to lysosomes, inducing the inevitable degradation of the GPCR [17].

## 3. Definition of Dopamine and Classification of Dopamine Receptors

Lipid rafts are involved in the regulation of dopamine receptors. Dopamine is a catecholamine that functions as a precursor of norepinephrine and epinephrine. However, during the past decades, it has been identified as a modulator of sodium balance and blood pressure by means of a renal dopaminergic system. Dopamine interacts with GPCRs that are classified into five genetically distinct types [3,4,5,18,19,20,21]: D_1_R, D_2_R, D_3_R, D_4_R, and D_5_R.

The dopamine receptors listed above are divided in two groups, D1-like and D2-like receptors, all of which are expressed in the kidney (Table 1). The D1-like receptors are coupled to G proteins Gs and Gq to stimulate adenylyl cyclase and phospholipase C activity, respectively; D_1_R and D_5_R belong to this group. The D2-like receptors are coupled to Gi and Go to inhibit adenylyl cyclase activity; D_2_R, D_3_R, and D_4_R belong to this group [3,4,5,18,19,20,21,22,23,24,25,26,27,28,29,30,31,32,33,34,35,36,37,38]. 

Dopamine receptors, like other GPCRs, have seven transmembrane domains, linked by three intracellular loops, three extracellular loops, an extracellular amino terminus, and an intracellular cytoplasmic C terminal tail [20,21]. Members of the same family of dopamine receptors (D1-like and D2-like) display considerable structural homology in terms of their transmembrane domains, NH2—terminal and COOH terminal domains [20,21].

However, the D1-like receptors have a short third intracellular loop whereas the D2-like receptors have a long third intracellular loop [20,21]. The NH2-terminal domain has the same number of amino acids in all the D1-like and D2-like receptors and contains a variable number of consensus *N*-glycosylation sites (Table 2) [8,20,21].

**Figure 10:**
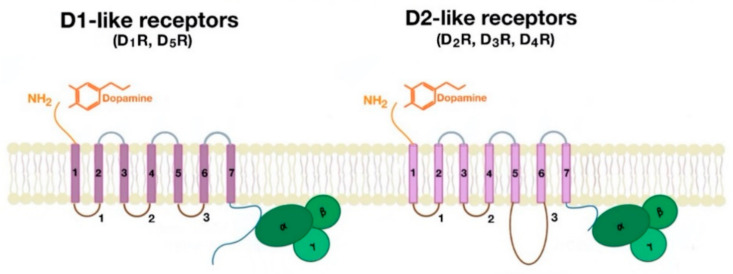
Structural differences between D1-like receptor (left side of picture) and D2-like receptors (right side of picture).

In the D1-like receptors, the COOH terminus is about seven times longer than that in D2-like receptors (Figure 10). The COOH terminus has abundant serine and threonine residues and contains a cysteine residue that is present in all GPCRs. In the D1-like receptors, the location of the cysteine residue is near the start of the COOH terminus. However, for the D2-like receptors, the COOH terminus ends with a cysteine residue [20].

## 4. Function of Renal D1-Like and D2-Like Receptors

A dysfunction in the production of intrarenal dopamine or dopamine receptor signaling can predispose to hypertension, with or without salt sensitivity that is related, in part, to dysregulation of the renin–angiotensin system [3,4,5,18,19]. Additionally, several studies indicate that renal dopamine and its receptors play a role in the reduction of reactive oxygen species production, inflammation, and the progression of CKD [3,5,19]. As the receptor signaling system depends on lipid rafts, failure to assemble and maintain the lipid raft, as well as to target the dopamine receptors to lipid rafts, would result in the aforementioned pathological states.

Dopamine receptors are important in establishing normal sodium balance and normal blood pressure under conditions of euvolemia and moderate volume expansion. Renal dopamine D1-like receptors cause renal vasodilation and natriuresis, the latter being due to inhibition of ion transport at the apical and basolateral membranes of renal tubules [3,4,5,18,19,22,23]. The natriuretic effect of dopamine is primarily exerted through D_1_R and D_5_R by inhibiting both the influx and efflux of Na^+^ in renal epithelial cells. This is accomplished by inhibiting multiple Na^+^ cotransporters, exchangers, and pump along the nephron, resulting in an increase in Na^+^ excretion [3,4,5,18,19,22,23].

Renal D2-like receptors also participate in the inhibition of ion transport during conditions of euvolemia and moderate volume expansion [3,5,18,19,22,23]. D_2_R, D_3_R, and D_4_R (D2-like receptors) are also involved in the CNS regulation of blood pressure; post-synaptic D2-like receptors increase blood pressure, while presynaptic D2-like receptors (the predominant action) produce the opposite effect [3,23].

## 5. Signaling Pathway of Renal Dopamine Receptors

Similar to other GPCRs, the dopamine signaling pathway is divided in four stages: (1) activation of dopamine receptor, (2) interaction of receptor with heterotrimeric G proteins, (3) signal transduction through effector molecules, and (4) cellular response. As with other GPCRs, the dopamine receptors need to be internalized, endocytosed, and retargeted to the lipid raft to be functional again. These processes cause desensitization and resensitization.

As previously mentioned, D1-like and D2-like receptors belong to the family of membrane receptors called GPCRs. GPCRs exert their actions via heterotrimeric G protein subunits α, β, and γ.

### 5.1. D1-Like Receptor Signaling Pathway

**Figure 11:**
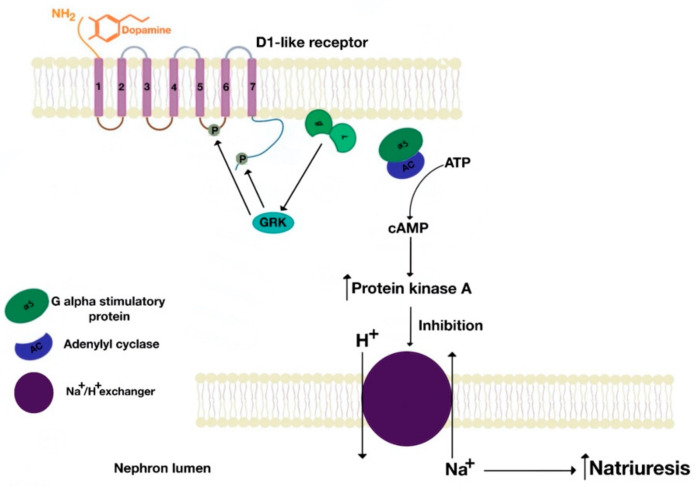
D1-like receptors are linked to Gαs subunit.

D1-like receptors are coupled with Gαs and Gαq [3,4,5,18,19,20,21,22,23,24,25]. The D1-like receptors, coupled with Gs, activate adenylyl cyclase, which breaks down ATP into cAMP (cyclic adenosine monophosphate). cAMP leads to the activation of protein kinase A, which functions as an effector molecule that inhibits the Na+/H+ exchanger type 3 (NHE3) at the brush border side of the proximal tubule and thick ascending limb of Henle [3,5,18,19,20,21,22,23,24,25,26] (Figure 11). However, NHE3 can be directly inhibited by Gsα, independent of protein kinase A, at least in renal proximal tubule brush border membranes. In these membranes, β/γ dimers can stimulate NHE3 activity [26]. The D_1_R, but not D_5_R, can couple to Go [27]. By contrast, D_5_R but not D_1_R can couple to Gz and Gα 12/13 [28,29].

**Figure 12:**
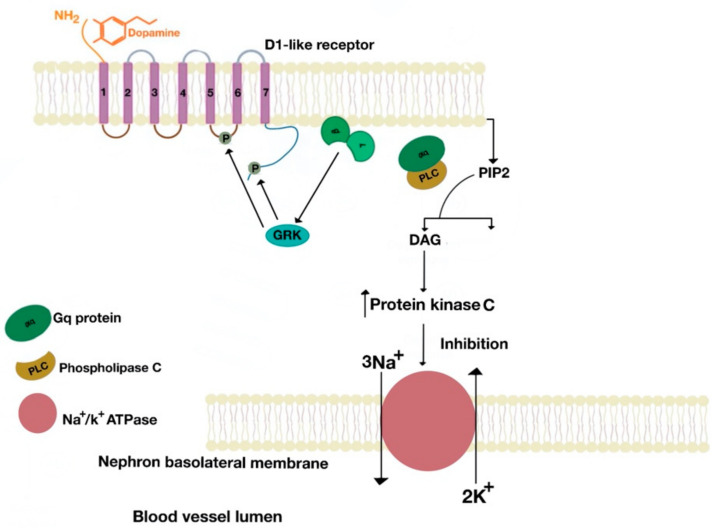
D1-like receptors are linked to Gαq subunit.

The D1-like receptors’ linkage to Gq activates phospholipase C, which in turn cleaves phosphatidylinositol 4,5-bisphosphate (lipid molecule found in the intracellular side of the cell plasma membrane, as well as in membranes of several intracellular organelles (C)) into DAG (diacylglycerol) and IP3 (inositol-1,4,5-trisphosphate). DAG activates protein kinase C (effector molecule) that ultimately inhibits Na^+^/K^+^-ATPase on the basolateral side of the nephron (Figure 12). The linkage of G protein subunits to D1-like receptors may be tissue-specific [30]. The effect of dopamine receptors on sodium transport is also tissue-specific. The D_5_R increases NHE3 activity in human embryonic kidney (HEK)293T cells [31].

### 5.2. D2-Like Receptor Signaling Pathway

The D2-like receptors, which consist of D_2_R, D_3_R, and D_4_R, couple to Gαi, which inhibits adenylyl cyclase and reduces cAMP production. D2-like receptors are also linked to Go [3,4,5,18,19,20,21,22,23,24]. D2-like receptors also inhibit calcium channel activity and modulate potassium channel activity [3,32,33,34]. D_2_R can couple to the same extent to Gαi and Gz but not to Gq11 or Gα12/13. There are two isoforms of D_2_R, the D_2_short and D_2_long; the latter is expressed in the kidney [35]. The D_2_short, via Rho A, can couple to phospholipase D [3,36]. In contrast to the other D2-like receptors, D_3_R linkage to Gαi is not robust and can actually be linked to Gαs and Gq11 [3,24,37,38].

## 6. Role of the D1-Like and D2-Like Receptors in Natriuresis and Antinatriuresis

Dopamine in the kidney plays a pivotal role in the inhibition of sodium, particularly in conditions of moderate sodium excess [3,4,5,18,19,22,24]. An increase in extracellular fluid increases renal tubular dopamine production and the converse decreases renal tubular dopamine production [3,39]. When extracellular fluid volume is decreased, the intravenous infusion of dopamine decreases sodium excretion, whereas when extracellular fluid volume is expanded, the intravenous infusion of dopamine increases sodium excretion [40]. Renal proximal tubule-selective deletion of dopamine synthesis impairs sodium excretion and increases blood pressure [19,41]. Long-term pharmacological blockade of dopamine receptor subtypes also decreases renal sodium excretion and increases blood pressure [3,18,19,22,24,42,43,44,45,46]. Moreover, inactivation of any dopamine receptor subtype gene in mice results in hypertension that may be salt-sensitive, depending on the genetic background [3,25,46]. The stimulation of D1-like receptors mediate natriuresis by inhibiting NHE3 (SLC9A3), sodium phosphate cotransporter (NaPi-IIa/SLC34A1 and NaPIIc/SLC34A3), Cl^−^/HCO_3_^−^ exchanger (SLC26A6) at the apical membrane, and electrogenic Na/HCO_3_ cotransporter (NBCe2, SLC4A5 [47,48]) and Na^+^/K^+^-ATPase at the basolateral membrane [3,18,19,23,25,49,50,51,52,53,54,55]. Currently, there are no commercially available agonists that can differentiate between the subtypes of D1-like receptors D_1_R and D_5_R [3]. Stepholidine is a D_1_R agonist and D_2_R/D_3_R antagonist [56]. LE-PM436 is a selective D_5_R antagonist [57]. The D_1_R and D_5_R interact to inhibit NHE3 and Na^+^/K^+^/ATPase activity via both the adenylyl cyclase and phospholipase C pathways [58]. In conscious rats, the renal interstitial infusion of D_1_R-specific antisense oligodeoxynucleotides impairs sodium excretion, providing evidence of the importance of the D_1_R in the inhibition of renal tubular sodium transport [49].

The effect of D2-like receptors on renal sodium transport is variable, including no effect, attenuation of sodium excretion, and increase in sodium excretion that may be related to the state of extracellular fluid volume [3,40]. Bromocriptine, a D2-like receptor agonist (D_2_R = D_3_R > D_4_R), as well as serotonin agonist increased Na^+^/K^+^/ATPase activity in rat renal proximal tubules [59]. The D_2_R agonist LY171555 increased Na^+^/K^+^/ATPase activity in Ltk-11 cells heterologously expressing the D_2_long receptor [60]. By contrast, the D_3_R agonist PD128907 inhibited Na^+^/K^+^/ATPase activity in rat renal proximal tubules [61]. The D_4_R agonist PD168077 also decreased the ability of insulin to stimulate Na^+^/K^+^/ATPase activity in rat renal proximal tubule cells [62]. Sulpiride (D_2_R = D_3_R > D_4_R), a D2-like receptor antagonist, impaired dopamine-mediated natriuresis in euvolemic males [63]. The D_3_R agonists PD128907 and 7-OH-DPAT increased sodium excretion in rats chronically fed a high-salt diet [61,64]. It is possible that activation of D2-like receptors may cause antinatriuresis or natriuresis in volume-depleted and volume-expanded states, respectively [25,40]. The role of specific D2-like receptors in this process needs to be clarified.

## 7. Renal D1-Like Receptors and Lipid Rafts

Lipid rafts are important in organizing signal transduction cascades [1,2,6,7,8,9,12,15,16,65,66]. D_1_R partitions to lipid as well as non-lipid rafts in human renal proximal tubule cells expressing wild-type GRK4 [67,68,69,70] and interacts with the lipid raft protein Cav1 [67,68,71,72,73,74,75] in renal proximal tubule cells. Additionally, it interacts Cav2 in HEK293 cells expressing D_1_R [76]. By contrast, in the presence of increased GRK4 activity, which is characteristic of GRK4 variants [46,77,78,79,80,81,82,83,84,85,86], in human renal proximal tubule cells, or increased renal GRK4 expression in rodents, the D_1_R may partition mainly to non-lipid rafts [87]. The D_5_R, as with the D_1_R, also partitions to lipid as well as non-lipid rafts in human renal proximal tubule cells [68], but in rat renal proximal tubule cells, the D_1_R and D_5_R are mainly expressed in non-lipid rafts [87]. However, GRK2, not GRK4, regulates the D_5_R [31]. Disruption of lipid rafts in human renal proximal tubule cells with methyl-β-cyclodextrin (β-MCD) [88] blocks the fenoldopam (D_1_-like receptor (D_1_R and D_5_R) agonist)-stimulated cAMP production [67]. β-MCD removes caveolar domains very efficiently but also non-specifically depletes plasma membrane cholesterol. Kidney-restricted disruption of lipid rafts in rats impairs D1-like receptor-mediated natriuresis and increases blood pressure [72].

As aforementioned, the SNX family plays a pivotal role in the sorting and transport of GPCRs through a series of endosomal compartments [89,90,91]. The first 20 min of D_1_R desensitization is in part due to SNX5 [69], and also to SNX19 (unpublished), and thereafter GRK4, and, to a lesser extent, GRK2 [84]. SNX5 is important in the recycling of glycosylated D_1_R to the plasma membrane that requires the di-leucine motif (L344–L345) at the D_1_R C-terminus [8]. The mutation of two specific palmitoylation sites (C347 and Y218) of a CRAC motif resulted in the absence of D_1_R in lipid rafts [70]. Some of the recycled D_1_R has to be targeted to lipid rafts to be functional; this is not afforded by SNX5, probably because it is mainly expressed in non-lipid rafts. The recycling of D_1_R to lipid rafts is probably due to SNX19, which is normally exclusively found in the lipid raft [70]. For D_1_R to function, a critical amount of D_1_R has to be inserted in lipid rafts where adenylyl cyclase 6 is expressed; adenylyl cyclase 6 is activated to a greater extent by D_1_R than D_5_R [67]. Adenylyl cyclase isoforms 2, 3, 6, 7, and 9 are expressed in the renal proximal tubule [92]. Acute siRNA (small interfering RNA)-mediated, renal-restricted depletion of SNX5 decreases sodium excretion and further elevates the already increased blood pressure of spontaneously hypertensive rats [69]. SNX5 and D_1_R are also important in the normal expression of insulin receptor in human renal proximal tubule cells [93].

As indicated above, SNX19, which is found exclusively in lipid rafts, participates in the early desensitization of D_1_R in human renal proximal tubules. siRNA-mediated silencing of SNX19 in human renal proximal tubule cells impairs the ability of the D_1_R/D_5_R agonist fenoldopam to inhibit renal sodium transport. Renal-restricted siRNA-mediated depletion of SNX19 in mice decreases D_1_R expression and increases oxidative stress and blood pressure [70,94]. Lipid raft bestows protection against oxidative stress by maintaining NOX (NADPH oxidase) in the inactive state in human renal proximal tubule cells [68], the opposite of what is observed in normotensive rat renal proximal tubule cells [87], thus the need for caution in extrapolating non-human studies to human physiology, pathophysiology, or pharmacology.

SNX1 is required for D_5_R trafficking following agonist stimulation. It strongly binds to D_5_R, but not to D_1_R [95]. Absence of SNX1 blunts the cAMP production via D_5_R and suppression of renal sodium transport. Renal-restricted, siRNA-mediated depletion of SNX1 in mice decreases sodium excretion and increases blood pressure [96]. SNX1^−/−^ mice also have increased blood pressure; the natriuretic effect of the D_1_R/D_5_R agonist fenoldopam is also impaired in these mice [96]. The impaired natriuretic effect of fenoldopam is associated with increased renal sodium transport by Na/K ATPase, as well as with other sodium cotransporters and exchangers. Interestingly, SNX1^−/−^ mice have increased oxidative stress; oxidative stress being the cause or effect of hypertension [16,18,19,96,97]. D_1_R and D_5_R dysfunction or deficiency increases blood pressure, in part by an increase in oxidative stress [3,4,5,16,18,19,70,97,98,99].

## 8. Renal D2-Like Receptors and Lipid Rafts

Renal D2-like receptors are also found in lipid rafts. However, there are not many studies that have explored the role and interaction between lipid rafts microdomains and each renal D2-like receptor. In mouse striatum and in particular HEK293T cells, heterologously expressing the D2Rlong, the D_2_R is in plasma membrane microcompartments that do not correspond to detergent-resistant lipid rafts; β-MCD does not increase the detergent solubility of D_2_R [100]. In Chinese hamster ovary cells heterologously expressing the adenosine A2A and D_2_Rlong, following agonist stimulation, they co-internalize with Cav1, which is expressed in lipid rafts [101]. However, in the rat frontal cortex, endogenous D_2_Rs are distributed in cytoplasmic, detergent-soluble, and detergent-resistant fractions but not in buoyant fractions, as is the case for D_1_R [102]. GPCRs, such as D1-like and D2-like receptors, need to link to β-arrestin for their sequestration/internalization. However, dopamine D_2_R and D_3_R have different interactions with arrestins; the D_3_R exhibits less translocation via arrestins than the D_2_R. Additionally, arrestin 3 preferentially binds to D_2_R over D_3_R [103]. Haloperidol, an antagonist to D_2_R, D_3_R, and D_4_R, as well serotonin receptors, can inhibit cholesterol biosynthesis and redistribute flotillin-1 in plasma membranes of SH-SY5Y neuroblastoma cells [104].

The C-terminal tail of D2-like receptors is shorter than those in D1-like receptors [20]. The C-terminal tails of the D2-like receptors are similar in their structure. All of them contain cysteine residues that have been shown to undergo palmitoylation to anchor the cytoplasmic tail of these receptors [105]. The palmitoylation is relevant for the functioning of the D_3_R and D_4_R. Different studies performed on D1-like receptors showed that S-palmitoylation or palmitoylation of cysteine residues in the C-terminal tail of these GPCRs aids with their targeting to the lipid raft and subsequently with their proper functioning [8,70].

As with D1-like receptors, SNXs are also involved in the regulation of D2-like receptors. SNX25 is expressed in many tissues, including the kidney [106]. The overexpression of SNX25 amplifies the expression of D_1_R and D_2_R. Additionally, it augments signaling mediated by D_2_R and disrupts the endocytosis and recycling of D_2_R [106]. However, it does not affect D_1_R desensitization [106,107]. SNX27 is involved in D_2_R activation of G protein-gated inwardly rectifying channels in the brain [108].

## 9. Conclusions

In summary, lipid rafts and microcompartments in lipid rafts are important in the function of GPCRs, including dopamine receptors. The dopamine receptor family is divided in two groups, all of which are expressed in the kidney, D1-like receptors (D_1_R and D_5_R) and D2-like receptors (D_2_R, D_3_R, and D_4_R). As the dopamine receptor signaling system depends on lipid rafts, failure to assemble and maintain the lipid raft, as well as to target the dopamine receptors to lipid rafts, results in impaired natriuresis and increased production of reactive oxygen species and inflammation, leading to essential hypertension and progression of CKD. D1-like receptors are coupled with Gαs and Gαq and D2-like receptors couple to Gαi and to Go [3,18,19,20,21,22,23,24,25,26,27,28,29,30,31,32,33,34,35,36,37,38]. The SNX family regulates the endocytosis, endosomal sorting, and endosomal signaling of dopamine receptors, targeting them to plasma membrane microdomains [89,90,91]. CRAC motif helps anchor the Cav isoforms and dopamine receptors to the plasma membrane [12,70,109]. S-palmitoylation or palmitoylation of cysteine residues in the C-terminal tail of dopamine receptors aids with their targeting to lipid rafts [8,70]. Agonist-induced activation of the receptor is followed by its subsequent internalization and re-insertion to the plasma membrane (desensitization and resensitization of the receptor), therefore restarting the dopamine receptor cycle. Further research on this topic can open new targets to stop the progression of hypertension and CKD, and their complications.

## Figures and Tables

**Table 1:** Transmembrane segments of D1-like and D2-like dopamine receptors.

	D_1_R and D_5_R	D_2_R and D_3_R	D_2_R and D_4_R
Amino acid sequence conservation within transmembrane domain	80%	75%	53%

**Table 2:** NH_2_ terminal tail in D1-like and D2-like receptors.

	D_1_R and D_5_R	D_2_R	D_3_R	D_4_R
Number of consensus *N*-glycosylation sites	2	4	3	1
